# A Spike Protein-Based Subunit SARS-CoV-2 Vaccine for Pets: Safety, Immunogenicity, and Protective Efficacy in Juvenile Cats

**DOI:** 10.3389/fvets.2022.815978

**Published:** 2022-03-14

**Authors:** Kairat Tabynov, Madiana Orynbassar, Leila Yelchibayeva, Nurkeldi Turebekov, Toktassyn Yerubayev, Nurali Matikhan, Tlektes Yespolov, Nikolai Petrovsky, Kaissar Tabynov

**Affiliations:** ^1^International Center for Vaccinology, Kazakh National Agrarian Research University, Almaty, Kazakhstan; ^2^Preclinical Research Laboratory With Vivarium, M. Aikimbayev National Research Center for Especially Dangerous Infections (NSCEDI), Almaty, Kazakhstan; ^3^T&TvaX LLC, Almaty, Kazakhstan; ^4^Central Reference Laboratory, M. Aikimbayev National Scientific Center for Especially Dangerous Infections (NSCEDI), Almaty, Kazakhstan; ^5^Vaxine Pty Ltd., Adelaide, SA, Australia; ^6^College of Medicine and Public Health, Flinders University, Adelaide, SA, Australia

**Keywords:** cats, SARS-CoV-2, COVID-19, vaccine, adjuvant, spike protein, delta variant

## Abstract

Whereas, multiple vaccine types have been developed to curb the spread of Severe Acute Respiratory Syndrome Coronavirus-2 (SARS-CoV-2) among humans, there are very few vaccines being developed for animals including pets. To combat the threat of human-to-animal, animal-to-animal, and animal-to-human transmission and the generation of new virus variants, we developed a subunit SARS-CoV-2 vaccine which is based on the recombinant spike protein extracellular domain expressed in insect cells and then formulated with appropriate adjuvants. Sixteen 8–12-week-old outbred female and male kittens (*n* = 4 per group) were randomly assigned into four treatment groups: spike protein alone; spike plus ESSAI oil-in-water (O/W) 1849102 adjuvant; spike plus aluminum hydroxide adjuvant; and a PBS control. All animals were vaccinated intramuscularly twice, 2 weeks apart, with 5 μg of spike protein in a volume of 0.5 ml. On days 0 and 28, serum samples were collected to evaluate anti-spike IgG, antibody inhibition of spike binding to angiotensin-converting enzyme 2 (ACE-2), neutralizing antibodies against wild-type and delta variant viruses, and hematology studies. At day 28, all groups were challenged with SARS-CoV-2 wild-type virus 10^6^ TCID_50_ intranasally. On day 31, tissue samples (lung, heart, and nasal turbinates) were collected for viral RNA detection, and virus titration. After two immunizations, both vaccines induced high titers of serum anti-spike IgG that inhibited spike ACE-2 binding and neutralized both wild-type and delta variant virus. Both adjuvanted vaccine formulations protected juvenile cats against virus shedding from the upper respiratory tract and viral replication in the lower respiratory tract and hearts. These promising data warrant ongoing evaluation of the vaccine's ability to protect cats against SARS-CoV-2 infection and in particular to prevent transmission.

## Introduction

Severe Acute Respiratory Syndrome Coronavirus-2 (SARS-CoV-2) is the pathogenic agent that causes the disease COVID-19. Although genetically closely related viruses have been isolated from *Rhinolophus* bats (horseshoe bat), the exact source of SARS-CoV-2 has yet to be established (1). Coronaviruses (CoVs) including SARS-CoV-2 have been shown to cross interspecies barriers and can infect feline populations, including domestic cats (*Felis catus*) (2).

There are an estimated 76 million pet dogs and 96 million pet cats living in ~70% of U.S. households (3). According to Mars Petcare, Russia's pet population includes 40.8 million cats and 22.6 million dogs, living in 59% of Russian households^1^. In respect of the ongoing COVID-19 pandemic, there is a risk that domestic or farmed animals could play a role in the maintenance and transmission of SARS-CoV-2. This could involve transmission from humans to animals, from animals to animals, and from animals to humans (4). Large epidemics of human-origin SARS-CoV-2 in farmed minks with subsequent transmission of mink-mutated strains back to humans have confirmed such a risk (5).

The role of cats is of particular interest, because they are in close contact with humans and frequently in contact with other cats. Case reports on cats living in the same household with COVID-19 patients indicated that the transmission may have occurred from humans to pets (6–8). As suggested in the literature on sporadic cases (9–12) and confirmed by experimental exposure (13–17), cats are susceptible to infection, can show mild clinical signs, and can transmit infection to other cats. Large cats (snow leopards, tigers, and lions) are also susceptible to infection, with deaths being reported in zoos. SARS-CoV-2 RNA was detected by RT-PCR in the lung, heart, kidney, liver, spleen, and intestine of infected cats with particularly high levels of infectious virus being recovered from the lung and heart (18). The estimated reproduction number R_0_ in cats was calculated to be higher than 1, suggesting cats could play a role in the transmission and maintenance of SARS-CoV-2. In the same studies (4), it is noted that levels of virus shedding in infected household cats were as high as those observed experimentally, with reported shedding levels as high as 10^8.5^ RNA copies per swab sample or RT-PCR Ct values as low as 21. Considering infected cats shed high levels of virus and droplet transmission is possible, the risk for cat-to-human transmission may be significant (4). Infected cats shed virus for prolonged periods, suggesting cats may play a role in viral epidemiology by transmitting the virus onwards, generating new variants, or acting as a virus reservoir (19).

While the global focus is on the human vaccine rollout, eradication of SARS-CoV-2 infections in animals could be an important part of virus control. According to the World Health Organization, vaccines may be useful to protect susceptible animal species and prevent generation and transmission of viral mutations (20). To combat the threat of human-to-animal, animal-to-animal, and animal-to-human transmission and the generation of new variants, we developed a subunit SARS-CoV-2 vaccine called NARUVAX-C19 (pets) based on the recombinant spike protein extracellular domain expressed in insect cells formulated with adjuvants.

In this study, we evaluated the safety, immunogenicity, and protective efficacy in juvenile cats of recombinant spike protein formulated with either alum or an oil-in-water (O/W) adjuvant. This was based on our experience in developing a human vaccine against COVID-19 (called NARUVAX-C19) (21), based on a similar formulation that provided complete protection and blocked virus transmission in hamster studies. The current study was the first to test the safety, immunogenicity, and protective efficacy of NARUVAX-C19 (pets) vaccine in juvenile cats.

## Materials and Methods

### Facility, Bioethics, and Biosafety Statement

All experiments with infectious SARS-CoV-2 were performed in biosafety level 3 (BSL-3) and animal biosafety level 3 (ABSL-3) facilities in the Central Reference Laboratory (CRL) of the M. Aikimbayev National Scientific Center for Especially Dangerous Infections (NSCEDI) of the Ministry of Health of the Republic of Kazakhstan (MoH RK), which was completed in 2017 and accredited according to ISO 35001:2019 Biorisk management for laboratories and other related organizations, by DEKRA (a German company and member of the international accreditation associations IAF and DAkkS) in 2020. The BSL-3 and ABSL-3 facilities used were designed by the standards outlined in *Biosafety in Microbiological and Biomedical Laboratories* (fifth edition). Features of the BSL-3 and ABSL-3 facilities include controlled access, entry/exit through a shower change room, effluent decontamination, negative air pressure, double-door autoclaves, gas decontamination ports, HEPA-filtered supply and double-HEPA-filtered exhaust air, double-gasketed watertight and airtight seals, and airtight dampers on all ductworks. The structure of the BSL-3 and ABSL-3 facilities was pressure-decay-tested regularly.

All activities inside the BSL-3 and ABSL-3 labs are monitored by trained guards via video cameras. Only authorized personnel who have received appropriate training can access the facility. Experienced personnel work in pairs in the facility. Staff wear powered air-purifying respirators that filter the air when they culture the virus and handle infected animals (e.g., juvenile cats); the researchers are disinfected before they leave the room and then shower on exiting the facility. The facility is secured by appropriate procedures approved by the NSCEDI institutional biosafety officers. All facilities, procedures, training records, safety drills, and inventory records are subject to periodic inspections and ongoing oversight by the institutional biosafety officers who consult frequently with the facility managers. The research program, procedures, occupational health plan, security, and facilities are reviewed annually by a MoH RK official.

The animal studies were carried out in accordance with the recommendations in the *Guide for the Care and Use of Laboratory Animals* (eighth edition). The protocol on bioethics was approved by the Institutional Animal Care and Use Committee of the NSCEDI of MOH RK (Approval Number 105/2021-04-19).

### Cells

Vero E6 cells were obtained from the American Type Culture Collection (Vero 76, clone E6, CRL-1586, ATCC) and maintained in Dulbecco's modified Eagle's medium (DMEM, Gibco) containing 10% fetal bovine serum (FBS, Gibco) and antibiotic/antimycotic (anti/anti, Gibco) solution and incubated at 37°C with 5% CO_2_.

### Virus

SARS-CoV-2 strain hCoV-19/Kazakhstan/KazNAU-NSCEDI-4635/2020 (GISAID accession no. EPI_ISL_1208952) belongs to the Wuhan variant with D614G and M153T mutations in the spike protein and was isolated from a nasopharyngeal swab in a 31-year-old man with COVID-19 infection in Almaty, Kazakhstan. We also used the delta variant (B.1.617.2) of SARS-CoV-2 isolated on August 17, 2021, from a nasopharyngeal swab of a COVID-19 patient in Zhanaozen, Kazakhstan. Both strains were isolated at the BSL-3 laboratory of the NSCEDI. The strains were passaged twice in Vero E6 cells, and stocks were frozen at −80°C in DMEM containing 4.5 g/L d-glucose, 1 mM sodium pyruvate, 2% FBS, 1% non-essential amino acids (Gibco), and 25 mM HEPES (Gibco). Virus stock was titrated on Vero E6 cells using the Reed and Muench method (22) based on eight replicates for dilution, and plaques were counted 72 h post infection to determine the 50% tissue culture infectious dose (TCID_50_) per milliliter.

### Recombinant Spike Protein Vaccine Design

The detailed methodology of obtaining recombinant spike protein ECD has been described previously (23). Briefly, the spike protein was identified from the SARS-CoV-2 genomic sequence in NCBI (access number: NC 045512) (24). The spike protein was modified by replacement of the transmembrane domain with a hexa-histidine tag, and the codon-optimized insect cell expression cassette was then cloned into pFASTBac1 and baculovirus generated according to standard Bac-to-Bac procedures. The recombinant baculovirus was multiplied in Sf9 cells until the third passage and then used to infect *Trichoplusia ni* (Tni) cells to express the spike protein ECD. After 72 h of infection, the cell culture supernatant was purified by centrifugation, and the recombinant ECD spike protein was purified on a HisTrap Excel column using an AKTA chromatography system, concentrated by ultrafiltration and replaced with PBS, sterilized by filtration. The sequence of the spike protein was confirmed by mass spectroscopy, SDS-PAGE gel, and western blotting. Endotoxin was detected with the PyroGene™ Endotoxin Detection System (Cat. No. 50-658U, LONZA, Walkersville, MD, USA), and residual DNA content in the final vaccine product was determined with the Quant-iT™ PicoGreen™ dsDNA Assay Kit (Thermo Fisher, P7589) according to manufacturers' instructions.

### Preparation of Vaccine Formulations

SARS-CoV-2 spike protein ECD was formulated with a squalene containing the O/W nanoemulsion-type adjuvant ESSAI O/W 1849102 (O/W Adj, SEPPIC, France) in a ratio of 50:50 (by volume) (21) or a wet gel suspension of aluminum hydroxide Alhydrogel^®^ adjuvant 2% (Alum Adj, InvivoGen, USA) at a ratio of 1:1 (by volume). Phosphate-buffered saline (PBS) was used as a negative control sample. All samples were confirmed to be sterile and contained <2 EU/ml endotoxin and <50 ng/ml residual DNA content.

### Experimental Design of Animal Studies

Sixteen 8- to 12-week-old outbred juvenile cats (females and males, each *n* = 8), weighing ~500–700 g, were obtained from a research colony maintained at the M. Aikimbayev National Scientific Center for Especially Dangerous Infections (NSCEDI; Almaty, Kazakhstan). Juvenile cats were screened negative for feline enteric coronavirus antibody prior to transfer. The negative SARS-CoV-2 status of the animals was confirmed by a commercial PCR kit (NSCEDI, Kazakhstan) after arrival of the animals to our facility. Animals were housed in 0.65 × 0.85 × 1.15 m cages in the facility with 35–45% humidity at 22–23°C and with air exchange at least 16 times per h.

The number of animals used was determined by following the “minimum-quantity-principle” in our protocol. Juvenile cats (*n* = 2 males and *n* = 2 females, per group) were assigned into four treatment groups to receive 5 μg of spike protein with or without alum or O/W adjuvant (Table 1). First and second doses were administered to respective juvenile cats intramuscularly in the right rear and left rear legs, respectively.

**Table 1:** Design of the experimental SARS-CoV-2 vaccine study.

**Group**	**No. of animals**	**Antigen**	**Adjuvant**	**Days of vaccination**	**Days of challenge/euthanasia**
1	4 (♀ 2, ♂ 2)	Spike 5 μg	-	0 and 14	28/31
2	4 (♀ 2, ♂ 2)	Spike 5 μg	ESSAI O/W 1849102	0 and 14	28/31
3	4 (♀ 2, ♂ 2)	Spike 5 μg	Alhydrogel^®^ adjuvant 2%	0 and 14	28/31
4	4 (♀ 2, ♂ 2)	Control (PBS)	-	0 and 14	28/31

On day 28 PFV, animals of all groups were lightly anesthetized with 11 mg/kg of ketamine hydrochloride (Dechra Veterinary Products, KS, US) by intramuscular injection and challenged with hCoV-19/Kazakhstan/KazNAU-NSCEDI-4635/2020 strain intranasally via a pipette into the nares (200 μl per nare) for a total volume of 400 μl; animals were observed until fully recovered from anesthesia. Virus back-titration was performed on Vero E6 cells immediately following inoculation, confirming that kittens received 10^6^ TCID_50_. All kittens were anesthetized on day 31 PFV (3 days post challenge) using ketamine (11 mg/kg), humanely euthanized (pentobarbital >80 mg/kg), and necropsied to collect tissue samples.

### Clinical Evaluations and Sample Collection

The cats were monitored at least once daily for at least 7 days for clinical signs (fever, anorexia, lethargy, respiratory distress, recumbency, coughing, sneezing, diarrhea/loose stool, vomiting, vocalization, injection site reactions (for ~30 min after each injection), injection site licking, and fever by a licensed veterinary practitioner. Body weights and temperatures (rectal) were documented daily every morning for 7 days after each vaccination and for 3 days after viral challenge.

Serum (in clot activator tubes) and whole-blood (in EDTA tubes) samples were collected from juvenile cats via leg venipuncture on days 0, 28, and 31. Serum samples were stored at −20°C until used in ELISA (anti-spike IgG), surrogate virus neutralization test (sVNT; ACE-2 cell receptor blocking), and virus neutralization test (VNT). Whole-blood samples were used immediately for blood cell counts. Oropharyngeal swabs soaked in DMEM were collected on days 0 and 3 post challenge. After collection, the swabs were placed in a tube containing 1 ml of DMEM with anti/anti solution and stored at −80°C. Prior to use for the viral RNA detection and virus titration assays, the swabs were vortexed for 1 min.

Necropsy tissue collection included lung, heart, and nasal turbinates. Tissues were halved and then placed into 1 ml tubes and frozen at −80°C or placed in standard tissue cassettes. Tissue homogenates were prepared by thawing tissue and placing 200 mg (±50 mg) of minced tissue in a tube containing 1 ml DMEM culture medium and chrome-steel beads (Biospec Products). Homogenization was performed with the TissueLyser II (Qiagen) for 30 s at 30 H and repeated two times. Supernatant was retained after centrifugation for viral RNA detection and virus titration.

### Blood Cell Counts

Complete blood cell counts were performed using fresh EDTA blood samples run on an automated HTI MicroCC-20 VET Veterinary Hematology Analyzer (High Technology Inc., North Attleboro, MA) according to the manufacturer's protocol using the HTI MicroCC-20 VET reagent pack and recommended calibration controls. Blood cell analysis included 20 parameters: white blood cells (WBCs), number of lymphocytes (LYM#), number of mid-sized cells (MID#; MID cells include less frequently occurring and rare cells correlating to monocytes, eosinophils, basophils, blasts, and other precursor white cells that fall in a particular size range), number of granulocytes (GRA#), percentage of lymphocytes (LYM%), percentage of mid-sized cells (MID%), percentage of granulocytes (GRA%), red blood cells (RBC), hemoglobin (HGB), mean corpuscular hemoglobin concentration (MCHC), mean corpuscular hemoglobin (MCH), mean corpuscular volume (MCV), red blood cell distribution width-coefficient of variation (RDW-CV), red blood cell distribution width-standard deviation (RDW-SD), hematocrit (HCT), platelet (PLT), mean platelet volume (MPV), platelet distribution width (PDW), plateletcrit (PCT), and platelet–large cell ratio (P-LCR). Two control samples (one normal and one abnormal) were included in each assay run.

### RNA Extraction and Reverse-Transcription Quantitative PCR (RT-qPCR)

SARS-CoV-2-specific RNA was detected using an RT-qPCR assay. Briefly, tissue homogenates (lung, heart, and nasal turbinates) and oropharyngeal swabs in DMEM were mixed with an equal volume of RLT RNA stabilization/lysis buffer, and 200 μl of sample lysate was then used for extraction using the QIAamp Viral RNA Mini Kit (Qiagen, Hilden, Germany) following the manufacturer's instructions. Briefly, 140 μl of each sample was mixed with 560 μl of buffer AVL containing carrier RNA and incubated for 10 min at room temperature. After addition of 560 μl of 100% ethanol, the samples were passed through purification columns by centrifugation. The columns were washed sequentially with 500 μl of buffer AW1 and 500 μl of buffer AW2, and RNA was eluted using 40 μl of RNase-free water. To quantify SARS-CoV-2 RNA levels, we used the commercial real-time RT-PCR kit (NSCEDI) according to the manufacturer's instructions. The following primer pairs targeting the N gene of the SARS-CoV-2 virus were used: F 5′-GGGGAACTTCTCCTGCTAGAAT; R 5′-CAGACATTTTGCTCTCAAGCTG. Amplification was performed as follows: 50°C for 10 min, 95°C for 2 min, then 45 cycles consisting of 95°C for 15 s, 60°C for 30 s, and a default melting curve in the Rotor-Gene^®^ machine (Qiagen, USA). When the Ct values (cycles) on the FAM/Green and JOE/Yellow channels were ≥40, the samples were considered negative for SARS-CoV-2.

### Anti-spike Binding IgG ELISA

Ninety-six-well microplates (Nunc MaxiSorp, #2297421, Invitrogen, USA) were coated with pre-titrated 0.5 μg/ml recombinant spike protein in a commercial buffer (ELISA Coating Buffer, #B288159, BioLegend) overnight. Plates were blocked using an ELISA Assay Diluent (#421203, BioLegend) at 200 μl per well and incubated under constant shaking (300–330 rpm on a PST-60HL thermal shaker, Biosan) for 1 h at room temperature. The plates were washed four times with ELISA Wash Buffer (# 421601, BioLegend). Juvenile cat serum samples were titrated twofold from dilutions 1:160–1:163,840, and 100 μl samples were added from each dilution to the wells and incubated under constant shaking (300–330 rpm) for 1.5–2 h at room temperature. After washing (×4), secondary goat Anti-Cat IgG Fc (HRP) biotinylated antibodies (1:10,000, #ab112801, Abcam, MA, USA) were added, and the plates were incubated (1 h at room temperature with shaking). After additional washing (×4), plates were incubated with HRP streptavidin (#405210, BioLegend, 1:1000, 100 μl per well) for 30 min at room temperature with shaking. Finally, plates were washed (five times) and added with a ready-to-use TMB substrate (#N301, Thermo Fisher Scientific, 100 μl per well). The color reaction was stopped by adding a stop solution (#B308260, BioLegend, 100 μl per well), and the optical density was measured (measuring wavelength 450 nm, reference wavelength 630 nm) on a Stat Fax 2100 analyzer (Awareness Tech). The cutoff value for determining the titer was calculated based on the average optical density (OD) values of the wells containing only the buffer (blank) + three standard deviations.

### sVNT

The SARS-CoV-2 sVNT Kit (L00847; GenScript, Piscataway, USA) was used according to the manufacturer's instructions to detect antibodies that inhibit the binding of spike RBD to human ACE2. Each sample was tested in duplicate. Briefly, samples and controls were incubated with horseradish peroxidase-conjugated RBD (HRP-RBD) at 37°C for 30 min. The mixtures were added to a hACE2-coated capture plate and incubated at 37°C for 15 min. The plates were then washed, removing the HRP-RBD neutralizing antibody complexes and allowing the unbound HRP-RBD and HRP-RBD non-neutralizing antibody complexes to remain bound to hACE2. The TMB solution was added and allowed to incubate at room temperature for 15 min, after which the reaction was stopped with a stop solution. The OD of each well was measured by spectrophotometry at 450 nm. The percentage of inhibition of the sample was calculated as (1–average OD of the sample/average OD of the negative control) ×100%. A sample with an inhibition percentage <30% was considered “negative” and one with an inhibition percentage ≥30% was considered “positive” for SARS-CoV-2-neutralizing antibodies. The following sVNT antibody levels were defined according to the level of inhibition: low (30–59%), medium (60–89%), and high (≥90).

### VNT

Serum samples were assayed for SARS-CoV-2-neutralizing antibodies at the BSL-3 laboratory of the CRL using a VNT (without the staining step) (25). Briefly, serum samples were heat-inactivated at 56°C for 30 min; then, 80 μl of three-fold serially diluted sera (for final dilutions with the virus of 1:10 to 1:7,290) was pre-incubated with 80 μl of 100 TCID_50_/ml of SARS-CoV-2 (hCoV-19/Kazakhstan/KazNAU-NSCEDI-4635/2020, the Wuhan variant with D614G and M153T mutations in the spike protein and hCoV-19/Kazakhstan/KazNARU-NSCEDI-5526/2021, the delta variant) in DMEM for 60 min at 37°C with 5% CO_2_. The 120 μl per well of virus-serum mixture was then cultured in duplicate on Vero E6 cells in 96-well plates (#3596, Corning™). After 60 min, all the virus–serum mixture was removed, and 100 μl of each respective serum dilution was added to the cells. Finally, 100 μl per well of DMEM containing 2% FBS, supplemented with anti/anti solution (Gibco) was added. The neutralization titers were determined at 5 days post infection. The titer of a sample was recorded as the reciprocal of the highest serum dilution that provided 100% neutralization of the reference virus, as determined by visualization of cytopathic effect (CPE). Samples were considered seropositive when a dilution of at least 1:10 reduced the formation of CPE.

### Virus Titration Assay

Confluent Vero E6 cells in 96-well plates were infected with 200 μl of 10-fold dilutions (10^−1^ to 10^−7^) of the tissue homogenates (lung, heart, and nasal turbinates) and oropharyngeal swabs. After a 60 min incubation, the virus inoculum was removed, the cells were washed once with PBS, and then DMEM with 2% FBS and anti/anti solution was added and incubated for 5 days at 37°C under 5% CO_2_. The plates were observed daily for the presence of CPE by means of an inverted optical microscope. The endpoint titers were calculated according to the Reed and Muench method (22) based on six replicates for titration. Virus titers are expressed as log_10_ TCID_50_/ml.

### Statistical Analysis

GraphPad Prism 9.0.0 (GraphPad Software, San Diego, CA, USA) was used for constructing graphs and statistical analysis of the experimental data. Differences in hematological parameters, antibody titers, viral load in swabs, and tissues between animal groups were assessed using Tukey's multiple-comparisons test or Dunnett's multiple-comparisons test. The detection limit of the infectivity titer was 1.2 log_10_ TCD_50_/ml. The detection limit of IgG titers was 1:125, and that of neutralizing antibodies was 1:10. For all comparisons, *P* < 0.05 was considered a significant difference. In the figures, ^*^P < 0.05; ^**^P < 0.01; ^***^P < 0.001, and ^****^P < 0.0001.

## Results

### Immunogenicity of Spike Protein-Based Vaccine Candidates in Juvenile Cats

**Figure 1:**
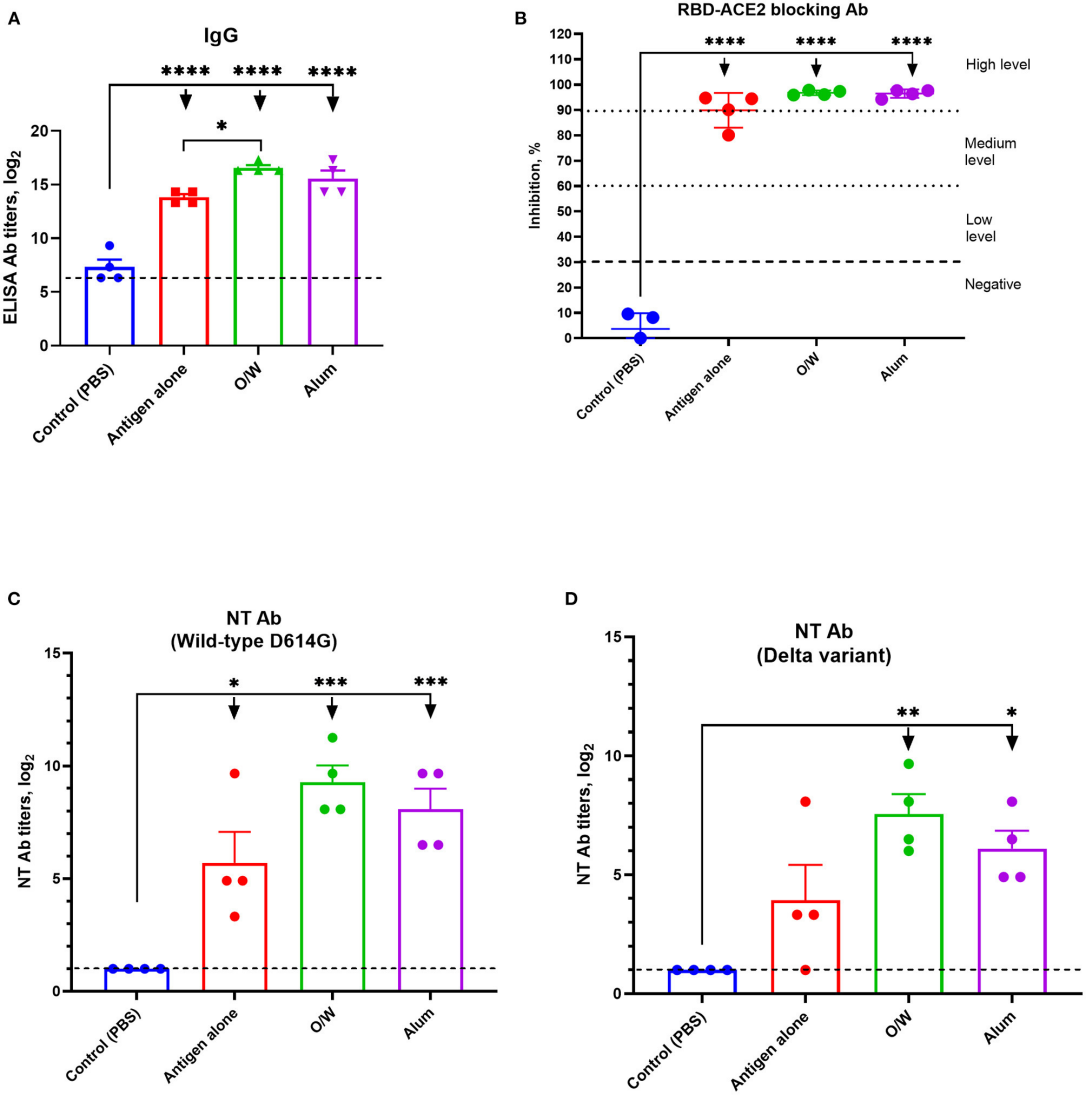
Spike-specific IgG **(A)**, RBD-ACE2 blocking antibody **(B)**, and neutralizing antibody **(C,D)** levels in juvenile cats 14 days after booster immunization. Viral neutralizing antibodies were assessed against wild-type D614G **(C)** and delta variant **(D)** viruses. Antigen was used at a dose of 5 μg with O/W and alum adjuvants. For comparison, 5 μg antigen was given without adjuvant and a negative PBS control. Differences between groups were assessed using Tukey's multiple-comparisons test. For all comparisons, *P* < 0.05 was considered a significant difference. *P < 0.05; **P < 0.01; ***P < 0.001; and ****P < 0.0001.

Immunogenicity in juvenile cats of our vaccine candidates was evaluated at day 14 after a booster intramuscular immunization. The O/W-adjuvanted spike protein induced significantly higher anti-spike IgG titers (GMT 97,420) than immunization with spike protein alone (GMT 14,480) (Figure 1A), and alum-adjuvanted spike protein gave intermediate results (GMT 48,710). Anti-spike IgG titers for all vaccine formulations were higher than PBS-injected control animals (GMT 160).

All juvenile cats (4/4) that received O/W-adjuvanted or alum-adjuvanted spike protein developed high levels (Figure 1B), whereas immunization with spike protein alone only induced medium to high levels, of RBD-ACE2 blocking antibody.

Next, sera were assessed for neutralizing activity against wild-type D614G or delta variant (Figures 1C,D). Wild-type D614G-neutralizing antibodies were highest for the O/W-adjuvanted (GMT 945) and alum-adjuvanted (GMT 450) vaccines vs. spike alone (GMT 220). Cross-neutralizing antibodies against the delta variant were detectable in 100% of cats that received O/W-adjuvanted (GMT 315) and alum-adjuvanted (GMT 150) spike, vs. 75% of animals that received spike alone. Overall, neutralizing antibody titers against the delta variant were around threefold lower than against the wild-type virus across all vaccine groups (Figure 1D).

### Safety and Efficacy Study of a Spike Protein-Based Vaccine Candidates in Juvenile Cats

#### Safety After Vaccination

Minor vocalization was observed in both control and vaccinated juvenile cats after the prime and booster dose administration. The only injection site reaction observed was mild swelling, which resolved within 24 h in one O/W adjuvant immunized animal. Transient fevers (39.5–39.7°C) that resolved within 24 h post injection were observed in all groups of vaccinated cats (spike alone = 1/4; O/W Adj = 2/4 and 1/4; Alum Adj = 2/4 and 1/4) after the first and second dose administration, respectively (Table 2).

**Table 2:** Frequency of immediate local reactions and transient fever within each treatment group.

**Vaccination**	**Adverse event**	**Control cats (*n* = 4)**	**Vaccinated cats (*****n*** **= 4)**		
			**Antigen alone**	**O/W Adj**	**Alum Adj**
Prime	Vocalization	1 (25)	1 (25)	2 (50)	1 (25)
	Injection site reaction	–	–	1 (25)	–
	Injection site licking	–	1 (25)	2 (50)	1 (25)
	Transient fever (39.5–39.7°C)	–	1 (25)	2 (50)	2 (50)
Booster	Vocalization	1 (25)	1 (25)	2 (50)	1 (25)
	Injection site reaction	–	–	–	–
	Injection site licking	–	–	–	–
	Transient fever (39.5–39.7°C)	–	1 (25)	1 (25)	1 (25)

*Data are n (%)*.

#### Clinical Signs After Vaccination and Challenge

**Figure 2:**
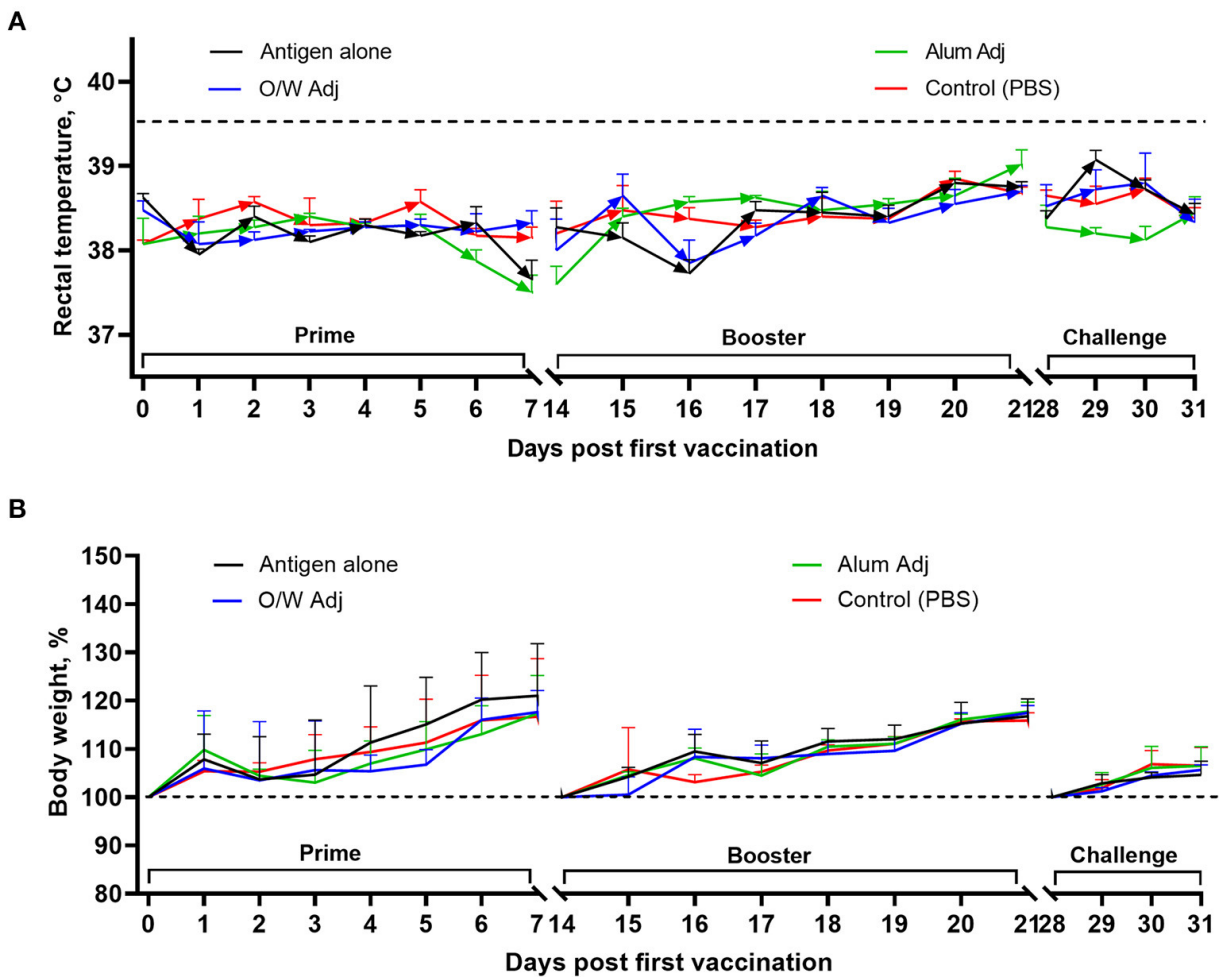
Rectal temperature **(A)** and body weight **(B)** in juvenile cats after vaccination and challenge. Antigen was used at a dose of 5 μg with O/W and alum adjuvants. For comparison, 5 μg antigen was given without adjuvant and a negative PBS control. A rectal temperature ≥39.5°C was considered as fever (dashed line). Data shown are mean ± SEM of four juvenile cats in each group.

Body temperature and clinical signs were recorded for 7 days after each vaccination and for 3 days after virus challenge. No remarkable abnormal clinical signs were observed after vaccination or challenge. Body temperatures of vaccinated and challenged (1 × 10^6^ TCID_50_ wild-type SARS-CoV-2 virus) juvenile cats remained normal (temperature <39.5°C) throughout the observation period (Figure 2A). Body weights of all juvenile cats increased throughout the study as expected for young animals without overt clinical disease (Figure 2B).

Complete blood counts were performed on days 0, 28, and 31 for the vaccinated and challenged animals. Overall, no significant changes in any blood cell parameters (WBC, LYM#, MID#, GRA#, LYM%, MID%, GRA%, RBC, HGB, MCHC, MCH, MCV, RDW-CV, RDW-SD, HCT, PLT, MPV, PDW, PCT, and P-LCR) were observed and counts remained within normal limits for most animals during the study. There was a significant increase in the mean corpuscular volume (MCV) in the control and spike alone vaccinated groups compared to the O/W- and alum-vaccinated groups of juvenile cats after challenge (Supplementary Table 1).

#### Viral Load in the Respiratory Tract and Cardiovascular System

Viral load was determined in oropharyngeal swabs and suspensions of nasal turbinates, heart, and lungs on day 3 after challenge by RT-qPCR (expressed in cycles/Ct) and viral titers by culture on Vero E6 cells (log_10_ TCID_50_/ml).

**Figure 3:**
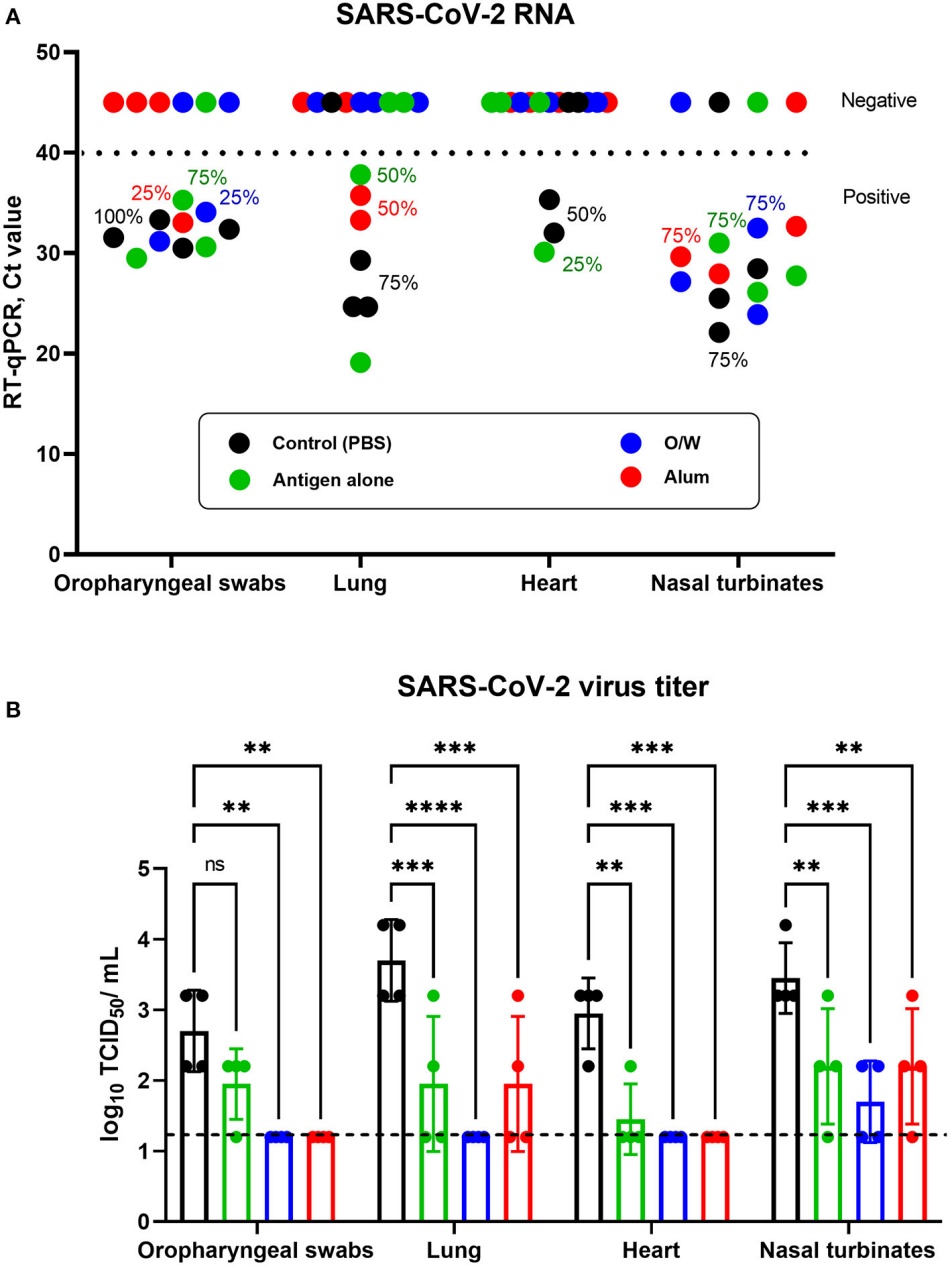
Viral load in the respiratory tract and cardiovascular system of juvenile cats. Antigen was used at a dose of 5 μg with O/W and alum adjuvants. For comparison, 5 μg antigen was given without adjuvant and a negative PBS control. Animals inoculated with SARS-CoV-2 virus were euthanized on day 3 after challenge, and their organs were collected for viral RNA detection and virus titration. Shedding and presence of viral RNA **(A)** and viral titers **(B)** in oropharyngeal swabs, lung, heart, and nasal turbinates. The horizontal dashed lines in **(A)** and **(B)** indicate the upper and lower limits of detection, respectively. Differences between groups assessed using Dunnett's multiple-comparisons test. For all comparisons, *P* < 0.05 was considered a significant difference. **P < 0.01; ***P < 0.001; and ****P < 0.0001.

In the control group, SARS-CoV-2 RNA was positive day 3 post-challenge by PCR in 4/4 oropharyngeal swabs (100%), 3/4 lungs (75%), 2/4 hearts (50%), and 3/4 nasal turbinates (75%). Similarly, in the spike-alone vaccine group, viral RNA was detected in 3/4 oropharyngeal swabs (75%), 2/4 lungs (50%), 1/4 hearts (25%), and 3/4 nasal turbinates (75%). By contrast, there was a complete absence of viral RNA (negative sample Ct of over 40) in heart samples in the groups that received O/W-adjuvanted and alum-adjuvanted spike vaccine and in lung samples of the O/W-adjuvanted groups (Figure 3A). However, detectable viral RNA by PCR was still seen in some oropharyngeal (25%) and nasal turbinate (75%) samples in the adjuvanted spike vaccine groups.

Infectious virus by CPE assay correlated with detection of viral RNA by PCR. Notably, infectious virus was not recovered from the oropharyngeal swabs, lungs, and heart samples in the O/W-adjuvanted and alum-adjuvanted spike vaccine groups. However, infectious virus was still detectable in nasal turbinates from some animals in the adjuvanted spike vaccine groups, although the virus titer was significantly lower than that in the control group (Figure 3B). These data show that the O/W-adjuvanted and alum-adjuvanted spike vaccines were highly efficient in blocking viral replication in the lower respiratory tract and heart but were unable to fully prevent infection in the upper respiratory tract. Overall, the O/W-adjuvanted vaccine was associated with the lowest viral loads across all tissues although this was not significantly different to the alum-adjuvanted vaccine.

## Discussion

The COVID-19 pandemic reminds us that zoonotic diseases pose major risks with 75% of all new human infections that have emerged over the past three decades being of animal origin (26). This underscores the importance of a “One Health” approach to disease control: the well-being of humans is inextricably linked to the health of the animals and the environment in which we are all living (27). Vaccination is an effective method of preventing a wide range of human and animal infectious diseases. Research into animal coronaviruses has led to several successful veterinary coronavirus vaccines and helps guide development of a safe and effective vaccine against SARS-CoV-2^2^. While the global focus remains on the human vaccine rollout, protection of animals could play an important role in pandemic recovery. While more than 190 COVID-19 vaccine candidates are in various stages of development (28), only two veterinary SARS-CoV-2 vaccines have been reported, Carnivac-Cov, an inactivated vaccine designed for carnivores (arctic foxes, cats, rats, and mink) (20) available in Russia (29), and an experimental recombinant spike protein vaccine produced by Zoetis that has been used in zoo animals in the USA^3^. An alphavirus replicon-based vaccine expressing the stabilized spike protein has also been reported to induce protective immunity and prevent transmission of SARS-CoV-2 between cats (19).

Our research team at the International Center for Vaccinology of the Kazakh National Agrarian Research University of the Ministry of Agriculture of the Republic of Kazakhstan (KazNARU) and NSCEDI in collaboration with Vaxine Pty Ltd, Australia, has been developing COVID-19 protein subunit vaccines for humans and animals. The vaccines are based on wild-type SARS-CoV-2 (Wuhan strain) spike protein ECD with a histidine tag to assist with purification and stabilization that is expressed in insect cells using the baculovirus transfection system. This spike protein ECD antigen when combined with Vaxine's proprietary Advax-CpG55.2 has previously been shown to be immunogenic, protective, and safe in mice and ferrets in an Australian-developed vaccine called COVAX-19 or SpikoGen (28) that has already successfully passed phase III clinical trials and in October 2021 received emergency use authorization for human adult use from the Iranian FDA (23). This same spike protein ECD when formulated with a squalene O/W (SWE) adjuvant (NARUVAX-C19 vaccine) provided complete protection of Syrian hamsters against SARS-CoV-2 infection and prevented transmission to naïve animals placed in the same cage as the challenged animals (21).

Adjuvants play an important role in ensuring that vaccine responses are robust and long-lasting (30, 31). In the current studies, we combined the spike ECD antigen with either Alhydrogel^®^ or the previously used O/W adjuvant. Alhydrogel is a wet gel suspension of aluminum oxyhydroxide that is the most commonly used human adjuvant (32, 33) and was included because a recent COVID-19 vaccine study using an alum-stabilized Pickering emulsion (PAPE) showed enhancement of RBD-specific IgG1 and IgG2a and IFN-γ-secreting T cells (31).

Spike ECD formulated with either alum or O/W adjuvants demonstrated no safety issues in juvenile cats. All vaccine formulations were well tolerated during prime and booster vaccinations, with only mild short-lived local reactions that included short-term vocalization and licking of the injection site. The only moderate injection site reaction occurred in a cat in the O/W-adjuvant group and was considered to be associated with the adjuvant, as O/W adjuvants are known to be locally reactogenic. Rectal temperatures after prime and booster vaccinations were not elevated, and the juvenile cats continued to gain weight throughout the study period. Hematological data showed no significant differences in the 20 measured hematologic parameters between vaccinated and control groups. Overall, all three vaccine formulations showed an acceptable tolerability and safety profile with a similar tolerability to an inactivated influenza virus vaccine in cats (34).

The data highlight the importance of adjuvants, in this case either O/W or alum, to vaccine immunogenicity (35). The adjuvanted vaccines and in particular the O/W-adjuvanted vaccine induced the highest overall spike-binding IgG, RBD-ACE2 blocking antibodies, and wild-type and delta variant-neutralizing antibodies. This translated into robust protection against challenge with a wild-type (D614G) virus. Notably, only the animals immunized with O/W-adjuvanted or alum-adjuvanted spike ECD had no recoverable infectious virus in day 3 oropharyngeal swabs, heart, and lung samples. This suggests a potential for our vaccine formulations, in addition to protecting like other COVID-19 vaccines against SARS-CoV-2 virus invasion into the lower respiratory tract and heart, to inhibit the release and transmission of virus to other animals.

Hematological parameters after challenge revealed a significant difference in the MCV between the PBS control and spike-alone injected groups on the one hand and the O/W- and alum-adjuvanted groups on the other. This is also consistent with the data from Curukoglu et al. (36) where a slight increase in MCV was also detected after challenge. This suggests that the severity of SARS-CoV-2 infection impacts the MCV.

Overall, the O/W-adjuvanted and alum-adjuvanted spike vaccines induced the highest neutralizing antibodies in juvenile cats, and this prevented infection of the lower respiratory tract and heart, although with only partial protection of the upper respiratory tract. The presence of infectious virus in our heart samples from the control juvenile cat is consistent with myocardial injury and fulminant myocarditis seen in some SARS-CoV-2-infected humans (37) and increased myocarditis previously reported in SARS-CoV-2-infected cats (38). Our data suggest, therefore, that spike protein may target the virus to the heart but that this effect can be blocked by neutralizing antibodies induced by our spike protein vaccine.

Our NARUVAX-C19 (pets) vaccine thereby joins other SARS-CoV-2 vaccines shown to provide protection in cats including the alum-adjuvanted inactivated Carnivac-Cov vaccine^4^ and a Venezuelan equine encephalitis virus replicon particle vaccine (19). In the case of our study, we applied a shorter immunization period with an interval of just 14 days between prime and booster vaccinations, compared to 21 days for the other vaccines. We also performed an earlier challenge at 14 vs. 21 days after booster vaccination. Notably, we showed our vaccine was able to cross-neutralize the delta variant, which has not been reported for these other vaccines.

Some limitations of this study need to be acknowledged. The small number of animals used per group was determined by animal welfare guidelines and the “minimum-quantity-principle”. Nevertheless, the results were highly consistent between the four cats in each group and between males and females within each group, with statistical differences seen when the adjuvanted vaccine groups were compared to saline and/or spike-alone immunized animals. Furthermore, the immunogenicity and protective efficacy seen in juvenile cats of the O/W-adjuvanted vaccine were consistent with the protection previously seen with the same vaccine formulation in Syrian hamsters (21). Another important limitation is the lack of long-term protection data. Future planned studies will explore further the effect of the vaccine on virus transmission and on protection against challenge with the delta virus variant, an important consideration given the recent appearance of highly transmissible delta strains in companion animals (39).

## Conclusion

In conclusion, the present research presents a comprehensive safety and efficacy evaluation in juvenile cats of a two-dose immunization regimen with a recombinant subunit spike protein vaccine with either of two commonly used adjuvants. Our data show such a vaccine is safe and well tolerated and able to provide strong protection to the lower respiratory tract and heart of juvenile cats, along with reducing viral loads in the upper respiratory tract. Considering these promising data, our vaccine will now be evaluated for its ability to protect against the delta variant and to prevent virus transmission, as well as the durability of protection over time before proceeding to large-scale animal field trials.

## Data Availability Statement

The original contributions presented in the study are included in the article/Supplementary Material, further inquiries can be directed to the corresponding author.

## Ethics Statement

The animal study was reviewed and approved by Institutional Animal Care and Use Committee of the NSCEDI of MOH RK (Approval Number 105/2021-04-19).

## Author Contributions

KaisT, TYes, and TYer: funding acquisition. KairT, MO, NT, and NM: investigation. KaisT, KairT, and MO: methodology. KaisT and KairT: project administration. KaisT: conceptualization, resources, software, and supervision. KairT and MO: data curation, validation, visualization, and writing—original draft. KaisT and NP: formal analysis and writing—review and editing. All authors contributed to the article and approved the submitted version.

## Funding

These studies were conducted at the expense of startup company T&TvaX LLC with the support of the Kazakh National Agrarian Research University and the M. Aikimbayev National Research Center for Especially Dangerous Infections. Development and supply of the spike protein antigen were supported in part by funding to NP from the National Institute of Allergy and Infectious Diseases of the National Institutes of Health under Contracts HHS-N272201400053C, HHSN272201800044C, and HHSN272201800024C; the MTPConnect Biomedical Translation Bridge Program; and a Fast Grant from George Mason University. The funders were not involved in the study design, collection, analysis, interpretation of data, the writing of this article or the decision to submit it for publication.

## Conflict of Interest

KaisT and KairT are affiliated with T&TvaX LLC which holds the rights to NARUVAX-C19 (pets) vaccine. NP is affiliated with Vaxine Pty Ltd. which holds the rights to Covax-19/Spikogen vaccine. The remaining authors declare that the research was conducted in the absence of any commercial or financial relationships that could be construed as a potential conflict of interest.

## Publisher's Note

All claims expressed in this article are solely those of the authors and do not necessarily represent those of their affiliated organizations, or those of the publisher, the editors and the reviewers. Any product that may be evaluated in this article, or claim that may be made by its manufacturer, is not guaranteed or endorsed by the publisher.

## Footnotes

^1^ https://zooinform.global/articles/pet-population-in-russia-increased-by-23/

^2^ https://coronavirusexplained.ukri.org/en/article/vdt0010/

^3^ https://news.zoetis.com/press-releases/press-release-details/2021/Zoetis-Donates-COVID-19-Vaccines-to-Help-Support-the-Health-of-Zoo-Animals/default.aspx

^4^ https://www.vidal.ru/veterinar/carnivac-cov-31130
