# Supplementary material for: A Spike Protein-Based Subunit SARS-CoV-2 Vaccine for Pets: Safety, Immunogenicity, and Protective Efficacy in Juvenile Cats
Source: Front Vet Sci. 2022 Mar 14;9:815978. doi: 10.3389/fvets.2022.815978 (PMC8967242; doi:10.3389/fvets.2022.815978)
Supplement: Supplementary file 1 [file Table_1.DOCX]

**Supplementary Material**

Table S1

Complete blood cell counts of juvenile cats after vaccination and challenge

| Parameter^1^ | Unit  (Reference ranges) | Group | Kittens (n=4 per group) | | |
| --- | --- | --- | --- | --- | --- |
|  |  |  | Day 0  (Mean ± SD) | Day 28  (Mean ± SD) | Day 31  (Mean ± SD) |
| WBC | 10^9^/L  (4,0-40,0) | Control (PBS) | 18,63 ± 4,79 | 21,75 ± 2,62 | 13,78 ± 2,61 |
|  |  | Antigen alone | 15,18 ± 4,86 | 20,10 ± 8,02 | 20,10 ± 8,02 |
|  |  | O/W Adj | 20,08 ± 6,22 | 18,60 ± 9,06 | 14,88 ± 1,98 |
|  |  | Alum Adj | 17,90 ± 4,07 | 19,25 ± 8,60 | 11,88 ± 3,42 |
|  |  | P Value (Max - Min) | 0,74 - 0,99 | 0,79 - 0.95 | 0,31 - 0,98 |
| LYM# | 10^9^/L  (1,5-7,0) | Control (PBS) | 5,29 ± 1,65 | 7,93 ± 1,40 | 3,30 ± 0,97 |
|  |  | Antigen alone | 4,40 ± 1,29 | 9,43 ± 6,25 | 6,10 ± 3,45 |
|  |  | O/W Adj | 5,30 ± 1,51 | 6,13 ± 2,93 | 4,50 ± 0,30 |
|  |  | Alum Adj | 4,53 ± 1,62 | 7,88 ± 3,44 | 5,98 ± 2,72 |
|  |  | P Value (Max - Min) | 0,94 - >0,99 | 0,68 - >0,99 | 0,35 - 0,87 |
| MID# | 10^9^/L  (0,0-1,9) | Control (PBS) | 0,80 ± 0,32 | 0,90 ± 0,07 | 0,35 ± 0,05 |
|  |  | Antigen alone | 0,95 ± 0,44 | 0,95 ± 0,51 | 0,80 ± 0,44 |
|  |  | O/W Adj | 0,80 ± 0,42 | 0,65 ± 0,30 | 0,63 ± 0,15 |
|  |  | Alum Adj | 1,05 ± 0,52 | 0,88 ± 0,43 | 0,45 ± 0,21 |
|  |  | P Value (Max - Min) | 0,63 - >0,99 | 0,63 - 0,99 | 0,20 - 0,96 |
| GRA# | 10^9^/L  (2,1-15,0) | Control (PBS) | 10,50 ± 2,33 | 12,93 ± 2,59 | 10,13 ± 1,86 |
|  |  | Antigen alone | 8,38 ± 1,80 | 9,65 ± 3,25 | 13,20 ± 5,04 |
|  |  | O/W Adj | 10,18 ± 2,42 | 11,83 ± 6,12 | 9,75 ± 2,35 |
|  |  | Alum Adj | 9,68 ± 2,42 | 10,50 ± 5,18 | 5,45 ± 1,35 |
|  |  | P Value (Max - Min) | 0,70 - 0,99 | 0,39 - 0,94 | 0,14 - 0,99 |
| LYM% | %  (12,0-45,0) | Control (PBS) | 37,48 ± 6,14 | 36,60 ± 6,43 | 23,70 ± 4,51 |
|  |  | Antigen alone | 33,08 ± 7,25 | 43,50 ± 10,68 | 29,25 ± 6,90 |
|  |  | O/W Adj | 32,00 ± 7,51 | 32,60 ± 5,13 | 30,10 ± 7,72 |
|  |  | Alum Adj | 39,08 ± 3,02 | 40,80 ± 5,11 | 35,93 ± 12,06 |
|  |  | P Value (Max - Min) | 0,58 - 0,97 | 0,40 - 0,77 | >0,05 - 0,46 |
| MID% | %  (2,0-9,0) | Control (PBS) | 4,58 ± 0,86 | 4,23 ± 0,76 | 4,43 ± 0,19 |
|  |  | Antigen alone | 4,30 ± 0,66 | 4,85 ± 0,78 | 3,85 ± 0,95 |
|  |  | O/W Adj | 3,90 ± 0,35 | 3,43 ± 0,37 | 4,38 ± 1,46 |
|  |  | Alum Adj | 4,45 ± 0,60 | 4,58 ± 0,98 | 3,50 ± 0,72 |
|  |  | P Value (Max - Min) | 0,48 - 0,99 | 0,35 - 0,86 | 0,24 - 0,99 |
| GRA% | %  (35,0-85,0) | Control (PBS) | 58,45 ± 3,15 | 59,18 ± 6,47 | 73,88 ± 4,70 |
|  |  | Antigen alone | 49,53 ± 6,61 | 51,65 ± 11,00 | 66,90 ± 7,81 |
|  |  | O/W Adj | 60,90 ± 3,98 | 63,98 ± 5,35 | 64,63 ± 8,47 |
|  |  | Alum Adj | 56,00 ± 5,18 | 54,63 ± 5,73 | 67,58 ± 12,49 |
|  |  | P Value (Max - Min) | 0,21 - 0,93 | 0,33 - 0,70 | 0,19 - 0,47 |
| RBC | 10^12^/L  (4,6-10,0) | Control (PBS) | 6,31 ± 0,86 | 5,44 ± 0,32 | 5,59 ± 0,23 |
|  |  | Antigen alone | 5,50 ± 0,46 | 5,54 ± 0,33 | 5,29 ± 0,56 |
|  |  | O/W Adj | 5,67 ± 0,42 | 5,59 ± 0,32 | 5,17 ± 0,45 |
|  |  | Alum Adj | 6,22 ± 0,29 | 5,48 ± 0,39 | 5,42 ± 0,66 |
|  |  | P Value (Max - Min) | >0,05 - 0,98 | 0,94 - 0,99 | 0,45 - 0,92 |
| HGB | g/L  (93,0-153,0) | Control (PBS) | 107,50 ± 5,02 | 105,75 ± 6,42 | 96,75 ± 5,31 |
|  |  | Antigen alone | 103,50 ± 5,68 | 100,00 ± 3,81 | 89,00 ± 6,28 |
|  |  | O/W Adj | 108,00 ± 8,15 | 101,25 ± 4,71 | 92,75 ± 5,12 |
|  |  | Alum Adj | 109,25 ± 12,21 | 101,00 ± 5,34 | 95,25 ± 12,19 |
|  |  | P Value (Max - Min) | 0,77 - 0,99 | 0,54 - 0,70 | 0,30 - 0,98 |
| MCHC | g/L  (300,0-380,0) | Control (PBS) | 330,75 ± 17,63 | 335,25 ± 11,39 | 308,75 ± 2,59 |
|  |  | Antigen alone | 336,50 ± 13,79 | 321,75 ± 28,95 | 302,75 ± 5,80 |
|  |  | O/W Adj | 341,75 ± 20,05 | 339,25 ± 19,14 | 314,50 ± 4,27 |
|  |  | Alum Adj | 343,25 ± 10,87 | 343,00 ± 22,56 | 299,00 ± 9,41 |
|  |  | P Value (Max - Min) | 0,55 - 0,91 | 0,49 - 0,96 | 0,71 - 0,91 |
| MCH | pg  (13,0-21,0) | Control (PBS) | 17,23 ± 0,90 | 18,15 ± 0,59 | 17,30 ± 0,41 |
|  |  | Antigen alone | 16,68 ± 1,93 | 18,15 ± 1,74 | 16,90 ± 0,73 |
|  |  | O/W Adj | 16,70 ± 0,79 | 18,18 ± 0,71 | 18,03 ± 0,86 |
|  |  | Alum Adj | 17,75 ± 0,87 | 18,68 ± 0,53 | 17,58 ± 0,35 |
|  |  | P Value (Max - Min) | 0,77 - 0,79 | 0,78 - >0,99 | 0,59 - 0,95 |
| MCV | fL  (39,0-52,0) | Control (PBS) | 50,78 ± 0,97 | 51,18 ± 1,13 | 61,30 |
|  |  | Antigen alone | 50,05 ± 1,30 | 50,55 ± 1,37 | 60,90 |
|  |  | O/W Adj | 49,40 ± 2,19 | 49,73 ± 2,09 | 50,38 |
|  |  | Alum Adj | 50,58 ± 1,15 | 51,08 ± 1,05 | 51,83 |
|  |  | P Value (Max - Min) | 0,38 - 0,99 | 0,34 - 0,99 | 0,95 (Control vs. Antigen alone)  <0,0001 (Control vs. O/W)  <0,0001 (Control vs. Alum) |
| RDW-CV | %  (14,0-18,0) | Control (PBS) | 15,48 ± 0,93 | 15,23 ± 0,55 | 13,60 ± 0,35 |
|  |  | Antigen alone | 15,40 ± 1,80 | 16,00 ± 2,42 | 13,63 ± 0,29 |
|  |  | O/W Adj | 15,53 ± 0,93 | 15,98 ± 1,14 | 13,65 ± 0,43 |
|  |  | Alum Adj | 14,75 ± 1,01 | 14,50 ± 0,95 | 12,93 ± 0,38 |
|  |  | P Value (Max - Min) | 0,67 - 0,99 | 0,64 - 0,67 | 0,73 - >0,99 |
| RDW-SD | fL  (35,0-56,0) | Control (PBS) | 37,35 ± 2,71 | 37,73 ± 1,38 | 35,05 ± 1,22 |
|  |  | Antigen alone | 37,23 ± 1,94 | 39,70 ± 1,49 | 34,43 ± 1,95 |
|  |  | O/W Adj | 36,50 ± 1,85 | 37,70 ± 1,22 | 35,55 ± 1,10 |
|  |  | Alum Adj | 37,08 ± 1,17 | 35,70 ± 1,59 | 34,70 ± 1,28 |
|  |  | P Value (Max - Min) | 0,80 - 0,99 | 0,20 - >0,99 | 0,90 - 0,98 |
| HCT | %  (28,0-49,0) | Control (PBS) | 35,78 ±2,63 | 34,30 ± 2,12 | 31,33 ± 1,60 |
|  |  | Antigen alone | 31,35 ± 3,89 | 31,28 ± 2,74 | 29,38 ± 1,89 |
|  |  | O/W Adj | 30,48 ± 1,06 | 29,88 ± 0,54 | 29,55 ± 1,44 |
|  |  | Alum Adj | 37,90 ± 2,40 | 35,40 ± 3,02 | 31,95 ± 4,50 |
|  |  | P Value (Max - Min) | 0,06 - 0,22 | >0,05 - 0,87 | 0,57 - 0,97 |
| PLT | 10^9^/L  (100,0-514,0) | Control (PBS) | 172,25 ± 32,92 | 175,25 ± 39,35 | 180,75 ± 84,25 |
|  |  | Antigen alone | 151,50 ± 15,26 | 144,25 ± 19,23 | 165,75 ± 35,49 |
|  |  | O/W Adj | 113,75 ± 7,85 | 133,00 ± 9,77 | 173,25 ± 37,99 |
|  |  | Alum Adj | 112,75 ± 7,63 | 130,25 ± 25,90 | 125,75 ± 34,85 |
|  |  | P Value (Max - Min) | 0,17 - 0,85 | 0,19 - 0,47 | 0,08 - 0,98 |
| MPV | fL  (5,0-11,8) | Control (PBS) | 7,28 ± 0,46 | 7,53 ± 0,48 | 7,98 ± 0,08 |
|  |  | Antigen alone | 7,20 ± 0,22 | 7,35 ± 0,36 | 7,90 ± 0,07 |
|  |  | O/W Adj | 7,55 ± 0,25 | 7,65 ± 0,52 | 8,05 ± 0,18 |
|  |  | Alum Adj | 7,25 ± 0,11 | 7,40 ± 0,10 | 7,73 ± 0,19 |
|  |  | P Value (Max - Min) | 0,43 - 0,99 | 0,72 - 0,89 | 0,49 - 0,97 |
| PDW | fL  (10,0-18,0) | Control (PBS) | 16,45 ± 1,17 | 18,05 ± 2,48 | 12,28 ± 2,76 |
|  |  | Antigen alone | 16,55 ± 0,71 | 18,00 ± 3,97 | 9,60 ± 0,14 |
|  |  | O/W Adj | 13,80 ± 3,36 | 12,95 ± 5,60 | 9,98 ± 1,76 |
|  |  | Alum Adj | 16,00 ± 2,00 | 17,80 ± 2,78 | 16,48 ± 4,35 |
|  |  | P Value (Max - Min) | 0,46 - >0,99 | >0,05 - >0,99 | 0,13 - 0,57 |
| PCT | %  (0,1-0,5) | Control (PBS) | 0,10 ± 0,00 | 0,13 ± 0,03 | 0,14 ± 0,07 |
|  |  | Antigen alone | 0,10 ± 0,00 | 0,11 ± 0,02 | 0,12 ± 0,08 |
|  |  | O/W Adj | 0,11 ± 0,00 | 0,08 ± 0,01 | 0,14 ± 0,03 |
|  |  | Alum Adj | 0,11 ± 0,00 | 0,08 ± 0,02 | 0,08 ± 0,03 |
|  |  | P Value (Max - Min) | 0,95 - >0,99 | 0,12 - 0,76 | >0,05 - >0,99 |
| P-LCR | %  (13,0-43,0) | Control (PBS) | 15,98 ± 1,23 | 11,48 ± 2,94 | 10,03 ± 1,87 |
|  |  | Antigen alone | 14,20 ± 1,00 | 8,65 ± 2,42 | 7,55 ± 1,20 |
|  |  | O/W Adj | 16,53 ± 2,46 | 11,63 ± 4,12 | 10,30 ± 1,22 |
|  |  | Alum Adj | 16,75 ± 2,53 | 13,05 ± 2,84 | 11,03 ± 2,66 |
|  |  | P Value (Max - Min) | 0,58 - 0,97 | 0,23 - 0,99 | 0,33 - 0,99 |
| ^1^ See Methods section for abbreviations. | | | | | |
